# Anxiety and Levodopa Equivalent Daily Dose Are Potential Predictors of Sleep Quality in Patients With Parkinson Disease in Taiwan

**DOI:** 10.3389/fneur.2019.00340

**Published:** 2019-04-16

**Authors:** Chun-Wei Chang, Jun-Yu Fan, Bao-Luen Chang, Yih-Ru Wu

**Affiliations:** ^1^Department of Neurology, Chang Gung Memorial Hospital at Linkuo Medical Center and Chang Gung University College of Medicine, Taoyuan, Taiwan; ^2^Department of Nursing, Chang Gung University of Science and Technology, Taoyuan, Taiwan; ^3^Department of Nursing, Linkou Chang Gung Memorial Hospital, Taoyuan, Taiwan

**Keywords:** sleep quality, Parkinson disease, anxiety, levodopa equivalent dose, working

## Abstract

**Background and Purpose:** Non-motor symptoms of Parkinson disease (PD) have a strong negative impact on the health-related quality of life (QoL) of patients with PD. Sleep disturbance is an important non-motor symptom because of its high prevalence. However, previous studies investigating the determinants of sleep quality in patients with PD have revealed inconsistent results. Our study evaluated the correlations between sleep quality in patients with PD and disease-related variables, medications used depression, anxiety, and QoL and identified the determinants of sleep disturbance in people with PD in Taiwan.

**Methods:** A total of 134 patients with PD were recruited from the outpatient clinic. We examined the correlations between the Parkinson disease sleep scale-2 (PDSS-2) scores and different variables, namely the Unified Parkinson Disease Rating Scale, Parkinson disease questionnaire, Beck Depression Inventory, and Beck Anxiety Inventory (BAI). Logistic regression analysis was used to assess the potential predictive variables for sleep quality in patients with PD.

**Results:** Among our participants, 47.8% were classified as poor sleepers (PDSS-2 = 15–60). Correlation analysis demonstrated that poor sleepers exhibited longer disease durations, higher levodopa equivalent daily doses (LEDDs), higher PD severity, more depression and anxiety symptoms, poorer QoL, more frequent unemployed status, higher hypnotics use, higher dependency for activities of daily living, more motor impairments, and more therapy-related complications. Logistic regression revealed that the LEDD was a significant predictive factor of sleep quality.

**Conclusion:** Poor sleepers constituted approximately half of our patients with PD. The participants experienced more favorable sleep if they were currently working. Increased PD duration, severity, depression or anxiety symptoms, and doses of dopaminergic therapy were significantly associated with poor sleep quality. Continued working, attempts to treat comorbid anxiety or depression, and avoidance of overdosage of dopaminergic treatments may improve sleep quality in patients with PD.

## Introduction

Non-motor symptoms of Parkinson disease (PD) have gained increasing research attention in recent years because they have a greater negative impact on quality of life (QoL) compared with motor disabilities (1) and exhibit a relatively poor response to dopaminergic therapy due to the diverse underlying mechanisms (2). Among them, sleep disturbance is a common symptom, with an estimated prevalence of 45–90% in the population with PD (3, 4). Sleep disturbance not only contributes to an increased occurrence of other non-motor symptoms of PD but is also associated with a decline in QoL (5). Extensive assessments of sleep disturbance and identification of causative factors may help improve QoL in patients with PD. Polysomnography is considered the gold standard for the diagnosis of sleep disorders (6, 7); however, it is often reserved for patients with severe symptoms because of its low accessibility and high cost. Scales are more suitable assessment tools in clinics or at the bedside. However, previous studies that have used sleep scales, including the Parkinson Disease Sleep Scale (PDSS) (8), Pittsburgh Sleep Quality Index (9), and Scales for Outcomes in PD-Sleep (3), revealed inconsistent predictive factors associated with sleep in PD. Moreover, these studies were mostly conducted in Western populations.

The Parkinson Disease Sleep Scale 2 (PDSS-2) is a revised version of the PDSS that has been shown to have fair sensitivity or specificity and good test–test reliability in validation studies (10–12). It includes 15 items on motor symptoms at night, PD symptoms at night, and disturbed sleep subscales. Both overall sleep impairment severity screening and detailed component analysis can be conducted using this scale.

Continued employment in patients with PD was known important both for general life satisfaction and help reducing the symptoms of PD (13, 14). Physical exertion caused by regular work may help to improve sleep in patients with PD. In this study, we assessed the correlations between sleep quality in PD obtained using PDSS-2 and the disease-related variables, current -working status, PD treatments, depression, anxiety, and QoL. In addition, we identified the determinants of sleep disturbances in patients with PD in Taiwan.

## Participants and Methods

### Participants

Participants who fulfilled the following inclusion criteria were recruited from the outpatient clinics by a movement disorder specialist (YRW): (1) satisfied the UK Parkinson Disease Society Brain Criteria (15) (2), aged between 20 and 90 years old, (3) Mini-Mental State Examination (Chinese version of MMSE) score ≥24, and (4) had regular follow-up visits every 1–3 month(s) at the medical center. Exclusion criteria were as follows: (1) secondary Parkinsonism due to an organic disorder or traumatic brain injury and (2) participants without a confirmed diagnosis of PD, with irregular follow-up visits, who are disorientated or unable to follow instructions, or who have other genetic or degenerative diseases.

The study protocol was approved by the Institutional Review Board of Chang Gung Memorial Hospital Ethics Committee (No. 101-2491B). A trained research assistant conducted the interviews immediately after the follow-up clinic visit. After providing informed consent, patients underwent a structured interview on their global cognitive function, sleep quality, emotional status, and QoL. Levodopa equivalent daily dosage (LEDD) was calculated for each participant according to the formula described by Tomlinson et al. (16). The duration of each interview was ~60 min. From March to December 2013, two hundred and twenty six patients clinically diagnosed as having PD were recruited. Among them, 58 patients were excluded for not meeting the inclusion criteria and 17 patients were excluded due to incomplete data. The final sample comprised 134 patients with PD.

### Assessments

The PDSS-2 and the 39-item Parkinson disease questionnaire (PDQ-39) were used to assess sleep quality and QoL in PD, which involved scoring on a 5-point Likert scale ranging from 0 (never) to 4 (always) (10, 17). The MMSE was used to screen for cognitive impairment (18). The Hoehn and Yahr staging (H&Y) and the Unified Parkinson Disease Rating Scale (UPDRS) were used to assess the disease stage and severity of PD symptoms (19, 20). The Beck Depression Inventory (BDI) and Beck Anxiety Inventory (BAI) were used to evaluate the symptoms of depression and anxiety, respectively (21, 22). Both the BDI and BAI were self-reported questionnaires with a 4-point Likert scale ranging from 0 (not at all) to 3 (severely). All the instruments were applied in validated Chinese versions.

### Statistical Analysis

The participant characteristics are presented as the mean ± standard deviation for continuous variables and as frequencies for categorical variables.

Differences in MMSE, H&Y, UPDRS, PDSS-2, PDQ-39, BDI, and BAI by demographics were examined using the independent *t*-test, one-way analysis of variance, and Scheffe *post-hoc* tests. Categorical variables among the different sleep quality groups were compared using chi-squared tests and Fisher exact tests. The correlations between participants' PDSS-2 scores and variables of interest were examined using Pearson correlation coefficients. A logistic regression analysis (enter method) was used to survey the potential predictive variables for sleep quality in patients with PD. A multi-collinearity analysis with variance inflation factors of <10 was conducted prior to exploration of the predictors. All tests were 2 tailed. A *P* < 0.05 was considered statistically significant. All analyses were performed using SPSS 22 (IBM SPSS Inc., Chicago, IL, USA).

## Results

### Participants' Disease-Related Characteristics

Among the 134 participants, more than half were men (85; 63.43%), with a mean age of 64.98 years (SD 9.19); age ranged from 41 to 87 years. Less than half of the participants were still working either part time or full time (65; 48.51%), and of those, 30 (22.39%) were ≥65 years old. The mean disease duration was 7.86 ± 5.55 years, and MMSE score was 27.41 ± 1.81. Among the participants, 108 (80.6%) had mild PD symptoms, with H&Y stages of 1–1.5. The mean scores of UPDRS, PDSS-2, and PDQ-39 were 39.48 ± 18.30, 18.36 ± 16.92, and 24.67 ± 15.25, respectively. The mean LEDD dose was 617.06 ± 454.73. Table 1 presents the characteristics of the participants.

**Table 1:** Characteristics of the participants (*N* = 134).

**Characteristics**	**Mean (±SD) or *n* (%)**	**Range (min–max)**
Age (years)	64.98 (±9.19)	41–87
Sex		
Male	85 (63.4%)	
Female	49 (36.6%)	
Work		
Yes	65 (48.51%)	
No	69 (51.49%)	
Onset age (years)	57.29 (±10.66)	29–85
Disease duration (years)	7.86 (±5.55)	0–23
MMSE	27.41 (±1.81)	24–30
H & Y stage	1.43 (±0.64)	1–4
1 & 1.5	108 (80.6%)	
2 & 2.5	20 (14.9%)	
3 & 4	6 (4.5%)	
UPDRS	39.48 (±18.30)	4–93
Part I	3.36 (2.32%)	0–13
Part II	12.93 (6.3%)	1–32
Part III	20.29 (11.03%)	2–58
Part IV	3.50 (3.22%)	0–18
BDI	12.69 (±9.76)	0–51
BAI	12.23 (±18.30)	0–45
PDSS-2	18.36 (±16.92)	1–55
Good sleepers (<15)	70 (52.2%)	
Poor sleepers (≥15)	64 (47.8%)	
PDQ-39	24.67 (±15.25)	0–87.71
LEDD (mg)	617.06 (±454.73)	0–2395

*MMSE, Mini-Mental State Examination; H&Y, Hoehn and Yahr scale; UPDRS, Unified Parkinson's Disease Rating Scale; BAI, Beck Anxiety Inventory; BDI, Beck Depression Inventory; PDSS-2, Parkinson's Disease Sleep Scale-2; PDQ-39, 39-item Parkinson's Disease Questionnaire; LEDD, Levodopa Equivalent Daily Dosage*.

### Comparison Between Good Sleepers and Poor Sleepers

Table 2 presents the differences in variables according to the PDSS-2 scores. The severity of sleep disturbance in patients with PD was classified on the basis of the cutoff value of 15 for the PDSS-2 questionnaire, ranging between good sleeper (0–14) and poor sleeper (15–60) (12). A total of 64 participants (47.8%) were classified as poor sleepers in the study sample.

**Table 2:** Differences in the participants' characteristics according to the PDSS-2 scores (*N* = 134).

	**Severity of PDSS-2**		***p***
	**Good sleepers (<15)**	**Poor sleepers (≥15)**	
	**M ± SD or *n* (%)**	**M ± SD or *n* (%)**	
Age (years)	64.66 ± 8.85	65.33(9.60)	
Disease duration	6.21 ± 4.75	9.66 ± 5.83	^***^
MMSE	27.39 ± 1.76	27.44 ± 1.88	
Sex			
Male	47(35.1)	38 (28.4)	
Female	23 (17.2)	26 (19.4)	
Onset age (years)			
<50	25 (18.7)	33 (24.6)	
≥50	45 (33.6)	31 (23.1)	
Work			^*^
Yes	40 (29.9)	25 (18.7)	
No	30 (22.4)	39 (29.1)	
H & Y stage			^***^a^^
1 & 1.5	65(48.5)	43(32.1)	
2 & 2.5	5(3.7)	15(11.2)	
3 & 4	0(0%)	6(4.5)	
BDI	8.84 ± 8.53	16.91 ± 69.33	^***^
Normal (0–13)	60 (44.8)	25 (18.7)	^***^a^^
Mild (14–19)	4 (3.0)	22 (16.4)	
Moderate to severe (20–63)	6 (4.5)	17 (12.7)	
BAI	8.13 ± 5.16	16.72 ± 9.90	^***^
Normal (0–7)	34 (25.4)	8 (6.0)	^***^
Mild (8–15)	30 (22.4)	27 (20.1)	
Moderate to severe (16–63)	6 (4.5)	29 (21.6)	
UPDRS	30.79(13.44)	48.98(18.26)	^***^
Part I	2.89(2.37)	3.86(2.17)	^*^
Part II	9.99(4.51)	16.14(6.56)	^***^
Part III	16.99(9.04)	23.91(11.92)	^***^
Part IV	2.07(2.09)	5.06(3.52)	^***^
PDSS-2 total	8.87 ± 3.68	28.56 ± 11.67	^***^
Motor	1.64 ± 1.55	7.49 ± 4.24	^***^
Night symptom	1.16 ± 1.18	6.34 ± 4.27	^***^
Disturbed sleep	6.07 ± 3.23	12.11 ± 3.39	^***^
PDQ-39	17.30 ± 10.90	32.73± 15.32	^***^
LEDD (mg)	416.50 ± 277.76	836.43 ± 508.38	^***^
Dopamine agonist (mg)	0.66 ± 0.98	0.88 ± 1.08	
Levodopa dose (mg)	257.09 ± 207.81	647.39 ± 491.49	^***^
Hypnotics Use			^*^
Yes	11(7.8)	22(15.6)	
No	61(43.3)	47(33.3)	

*M, Mean; SD, standard deviation; PDSS-2, Parkinson Disease Sleep Scale-2; MMSE, Mini-Mental State Examination; BDI, Beck Depression Inventory; BAI, Beck Anxiety Inventory; PDQ-39, 39-item Parkinson Disease Questionnaire; LEDD, Levodopa Equivalent Daily Dosage*.

* *p < 0.05*,

*** *p < 0.001*.

a *Chi-squared test with Fisher exact test*.

The Student *t*-test and chi-squared test with the Fisher exact test were used for examining the differences in the characteristics between good sleepers (PDSS-2 < 15) and poor sleepers (PDSS-2 ≥ 15). The results revealed significant differences in work status (*P* = 0.036), disease duration (*P* < 0.001), BDI (*P* < 0.001), BAI (*P* < 0.001), PDQ-39 (*P* < 0.001), UPDRS (*P* < 0.001), LEDD (*P* < 0.001), levodopa dose (*P* < 0.001), and proportion of participants using hypnotics (*P* < 0.05). The subscales of UPDRS parts I-IV demonstrated significant differences in PDSS type (*P* < 0.001) as well as the subscales of PDSS-2 motor symptoms (*P* < 0.001), night symptoms (*P* < 0.001), and disturbed sleep scores (*P* < 0.001). No difference was observed in the dopamine agonist dose, sex, onset before 50 years of age, and current age of the participants.

### Correlation Analyses

The results of the correlation analyses revealed that PDSS-2 score was significantly and positively correlated with disease duration (γ = 0.33, *P* < 0.01), LEDD (γ = 0.41, *P* < 0.01), H&Y stage (γ = 0.38, *P* < 0.01), BAI (γ = 0.44, *P* < 0.01), BDI (γ = 0.39, *P* < 0.01), and PDQ-39 (γ = 0.53, *P* < 0.01) as well as with the UPDRS (γ = 0.44, *P* < 0.01), UPDRS part II (γ = 0.47, *P* < 0.01), UPDRS part III (γ = 0.27, *P* < 0.01), and UPDRS part VI (γ = 0.40, *P* < 0.01) scores. Thus, participants with longer disease durations, higher LEDDs, higher PD severity, more depression and anxiety symptoms, poorer QoL, more PD symptoms, higher dependency for activities of daily living, more motor impairments, and more therapy-related complications demonstrated poorer sleep quality. Similar results were obtained in the subscales of PDSS-2 motor symptoms at night, PDSS-2 symptoms at night, and PDSS-2 disturbed sleep. Table 3 lists the interrelationships among these variables.

**Table 3:** Correlations among variables (*N* = 134).

	**PDSS-2 total**	**PDSS-2 motor|**	**PDSS-2 Night symptom**	**PDSS-2 Disturbed sleep**
Onset age	−0.12	−0.20^*^	−0.19^*^	−0.06
Current age	0.06	−0.07	0.05	0.07
Disease duration	0.33^**^	0.27^**^	0.26^**^	0.25^**^
MMSE	0.05	0.01	−0.03	−0.07
UPDRS total	0.44^**^	0.38^**^	0.45^**^	0.40^**^
UPDRS Part I	0.17	0.15	0.17^*^	0.21^*^
UPDRS Part II	0.47^**^	0.43^**^	0.47^**^	0.42
UPDRS Part III	0.27^**^	0.23^**^	0.28^**^	0.21^*^
UPDRS Part IV	0.40^**^	0.40^**^	0.44^**^	0.43^**^
BDI	0.39^**^	0.25^**^	0.40^**^	0.47^**^
BAI	0.44^**^	0.39^**^	0.41^**^	0.34^**^
H&Y	0.38^**^	0.25^**^	0.22^*^	0.25^**^
PDQ-39	0.53^**^	0.41^**^	0.52^**^	0.47^**^
PDSS-2	–	0.69^**^	0.69^**^	0.68^**^
LEDD total	0.41^**^	0.31^**^	0.37^**^	0.38^**^
Dopamine agonist	−0.01	−0.04	0.13	−0.04

*PDSS-2, Parkinson Disease Sleep Scale-2; UPDRS, Unified Parkinson Disease Rating Scale; BDI, Beck Depression Inventory; BAI, Beck Anxiety Inventory; H&Y, Hoehn and Yahr scale; PDQ-39, 39-item Parkinson Disease Questionnaire; LEDD, Levodopa Equivalent Daily Dosage*.

* *p < 0.05*,

** *p < 0.01*.

### Regression Analyses

All the variance inflation factors measured <10 (range: 1.36–3.68), indicating that the potential regression model did not violate the assumption of the regression model.

#### PDSS-2 as a Dependent Variable

The PDSS-2 scores were regressed against various potential predictors, namely disease duration, work status, H&Y stage, LEDD, and scores on the PDQ-39, BAI, BDI, and UPDRS parts II–IV. The overall model achieved statistical significance [*F*_(10, 123)_ = 7.55, *P* < 0.001 and γ2 = 0.38 (adjusted γ2 = 0.33)]. The H&Y stage had a significant effect on sleep quality in these [B = 3.88, *P* = .036, and 95% confidence interval (CI) = 0.25–7.51] after adjustment for the rest of the 9 covariates; that is, the higher the PD severity stage, the poorer the sleep quality. The proportion of variance (γ2) explained using the regression model amounted to 389% (adjusted 33%).

#### PDSS-2 Motor Symptoms at Night, PDSS-2 Symptoms at Night, and PDSS-2 Disturbed Sleep as Dependent Variables

The PDSS-2 motor scores were regressed against various potential predictors, namely age of onset, disease duration, H&Y stage, LEDD, and scores on the PDQ-39, BAI, BDI, and UPDRS parts II–IV. The overall model achieved statistical significance [*F*_(10, 122)_ = 4.78, *P* < 0.001 and γ^2^ = 0.28 (adjusted γ^2^ = 0.22)]. However, we were unable to identify any significant factor.

The PDSS-2 symptoms at night scores were regressed against various potential predictors, namely age of onset, disease duration, H&Y stage, LEDD, and scores on the PDQ-39, BAI, BDI, and UPDRS parts I–IV. The overall model achieved statistical significance [*F*_(11, 122)_ = 6.96, *P* < 0.001 and γ^2^ = 0.39 (adjusted γ^2^ = 0.33)].

After adjustment for the remaining 10 covariates, the PDQ-39 scores had a significant effect on the sleep quality in the night symptom subscale of patients with PD (B = 0.087, *P* = 0.015, and 95% CI = −0.017 to 0.156); that is, the poorer the QoL reported, the poorer the sleep quality in the night symptom subscale. The proportion of variance (γ^2^) explained using the regression model amounted to 39% (adjusted 33%).

The PDSS-2 disturbed sleep scores were regressed against various potential predictors, namely disease duration, work status, H&Y stage, LEDD, and scores on the PDQ-39, BAI, BDI, and UPDRS parts I–IV. The overall model achieved statistical significance [*F*_(11, 122)_ = 6.86, *P* < 0.001 and γ^2^ = 0.38 (adjusted γ^2^ = 0.33)]. Both UPDRS part VI and BDI were identified as significant predictors of sleep quality disturbances in patients with PD. The UPDRS part VI score had a significant effect on sleep quality disturbances in patients with PD (B = 0.40, *P* = 0.004, and 95% CI = 0.133 to 0.662) after adjustment for the remaining 10 predictors (covariates); that is, the higher the UPDRS part IV scores (more therapy-related complications), the greater the sleep quality disturbances.

After adjustment for the remaining 10 covariates, the BDI scores had a significant effect on the sleep quality disturbances in patients with PD (B = 0.14, *P* = 0.006, and 95% CI = −0.039 to 0.234); that is, the higher the level of depression reported, the greater the sleep quality disturbances. The proportion of variance (γ^2^) explained using the regression model amounted to 38% (adjusted 33%).

Table 4 presents the predictors of the PDSS-2, PDSS-2 motor problems, PDSS-2 symptoms at night, and PDSS-2 disturbed sleep.

**Table 4:** Predictors of PDSS-2, PDSS-2 motor symptoms, PDSS-2 night symptoms, and PDSS-2 disturbed sleep in the multiple regression analysis (*N* = 134).

	**PDSS-2**			**PDSS-2 Motor**			**PDSS-2 Night symptoms**			**PDSS-2 disturbance sleep**		
	**B**	**SE**	***p***	**B**	**SE**	***p***	**B**	**SE**	***p***	**B**	**SE**	***p***
Constant	1.214	3.280	0.712	3.398	2.645	0.201	2.576	2.302	0.265	5.692	1.137	0.000
Work status	–0.392	2.142	0.855	–	–	–	–	–	–	–1.379	0.741	0.065
Onset age	–	–	–	-0.054	0.041	0.197	–0.051	0.036	0.156	–	–	–
Disease duration (years)	–0.043	0.217	0.844	–0.072	0.091	0.430	–0.139	0.079	0.082	–0.107	0.075	0.154
H&Y stage (1–4)	3.883	1.833	0.036	0.468	0.657	0.477	–0.095	0.575	0.869	0.096	0.637	0.881
LEDD (mg)	0.003	0.003	0.222	0.000	0.001	0.771	0.001	0.001	0.241	0.001	0.001	0.166
UPDRS Part I	–	–	–	–	–	–	–0.252	0.147	0.090	–0.218	0.165	0.188
UPDRS Part II	0.157	0.272	0.564	0.177	0.099	0.076	0.135	0.085	0.116	0.106	0.094	0.261
UPDRS Part III	–0.070	0.120	0.559	–0.031	0.043	0.467	0.017	0.037	0.656	–0.009	0.042	0.826
UPDRS Part IV	0.546	0.387	0.161	0.206	0.140	0.144	0.226	0.121	0.063	0.398	0.134	0.004
BDI	0.029	0.140	0.837	–0.041	0.050	0.407	0.048	0.044	0.276	0.137	0.049	0.006
BAI	0.123	0.156	0.430	0.055	0.056	0.330	–0.016	0.048	0.741	–0.057	0.054	0.291
PDQ-39	0.224	0.116	0.056	0.053	0.041	0.193	0.087	0.035	0.015	0.037	0.040	0.355
	R^2^	0.38		R^2^	0.28		R^2^	0.39		R^2^	0.38	
	Adjusted R^2^	0.33		Adjusted R^2^	0.22		Adjusted R^2^	0.33		Adjusted R^2^	0.33	

*B, unstandardized regression coefficient; SE, Standard Error; H&Y, Hoehn and Yahr scale; UPDRS, Unified Parkinson Disease Rating Scale; BAI, Beck Anxiety Inventory; BDI, Beck Depression Inventory; PDSS-2, Parkinson Disease Sleep Scale-2; PDQ-39, 39-item Parkinson Disease Questionnaire; LEDD, Levodopa Equivalent Daily Dosage*.

#### Logistic Regression

Good or poor sleep was considered a dependent variable and was regressed against various potential predictors, namely disease duration, work status, H&Y stage, LEDD, and scores on the PDQ-39, BAI, BDI, and UPDRS parts I–IV. The overall model achieved statistical significance, with Wald χ^2^ = 68.296 and *P* < 0.01. The LEDD had significant effects on the sleep quality of patients with PD, with Wald χ^2^ = 6.80, *P* = 0.009 (OR = 1.002, 95% CI: 1.001 to 1.004). With an increase of 1 mg of LEDD, the patients with PD exhibited 1.002-fold higher risk of poor sleep quality. We also found the level of anxiety nearly reach statistically significant (*P* = 0.055) with Wald χ^2^ = 3.674, *P* = 0.055 (OR = 1.082, 95% CI: 0.998 to 1.172) (Table 5).

**Table 5:** Logistic regression showed BAI and LEDD had significant effects on the type of sleep quality.

**Variables**	**B**	**S.E**.	**Wals**	***P***	**OR**	**95% CI**	
						**Lower**	**Upper**
Constant	−4.466	0.853	27.396	0.000	0.011		
Disease duration (years)	−0.045	0.052	0.761	0.383	0.956	0.863	1.058
Work status	−0.275	0.526	0.274	0.601	0.759	0.271	2.130
H&Y stage (1–4)	0.064	0.481	0.018	0.894	1.066	0.415	2.36
PDQ39	0.031	0.032	0.964	0.326	1.032	0.969	1.098
UPDRS	0.039	0.021	3.456	0.063	1.040	0.998	1.084
LEDD (mg)	0.002	0.001	6.801	0.009	1.002	1.001	1.004
BDI	0.021	0.034	0.372	0.542	1.021	0.955	1.092
BAI	0.079	0.041	3.674	0.055	1.082	0.998	1.172

*H&Y, Hoehn and Yahr scale; PDQ-39, 39-item Parkinson's Disease Questionnaire; UPDRS, Unified Parkinson's Disease Rating Scale; LEDD, Levodopa Equivalent Daily Dosage; BAI: Beck Anxiety Inventory; BDI: Beck Depression Inventory*.

## Discussion

Our study demonstrated that patients with PD with poor sleep quality exhibited longer disease durations, higher H&Y stages, more severe PD symptoms, higher levodopa doses, and poor QoL. Sleep disturbance is an important non-motor symptom of PD, and its prevalence and severity increase with disease evolution (23, 24). It may also serve as a predictor of other non-motor symptoms and is detrimental to QoL in patients with PD (5). In previous studies, PDSS-2 has been correlated with H&Y stages, PDQ-39 index, UPDRS scores, BDI, LEDD, and PDSS-2 domain scores (motor, disturbed sleep, and night symptoms) (10, 12). Our study demonstrated consistent findings in these aspects. Poor sleepers exhibited higher proportions of hypnotic use, which was probably related to a higher need to relieve sleep disturbances compared with the good sleepers.

The present study revealed significant associations between PDSS-2 and higher levels of depression and anxiety. The regression analysis suggested depression symptoms as a predictor of PDSS-2 disturbed sleep component score. Depression and anxiety have been reported to be associated with poor sleep and to prominently contribute to poor QoL in PD (3, 25). Some studies have suggested that anxiety is a comorbidity of depression in PD (26, 27). By contrast, our study demonstrated that depression and anxiety symptoms are independent and potential predictors of the PDSS-2 disturbed sleep subscale and overall PDSS-2 scores. Studies have reported that depression is associated with subjective insomnia and deterioration of several sleep scales in patients with PD (3, 28–30). However, the presence of sleep disorders in patients with PD predicted more non-motor symptoms, including depression (5). This mutual relationship suggests a common mechanism underlying depression and sleep disturbances in patients with PD. Our study supports this perspective by demonstrating that depression specifically predicted the PDSS-2 disturbed sleep component score, but not the motor and night symptom component scores in PD. Despite the limited biological evidence, dopaminergic pathway dysfunction is the most likely contributor to both depression and sleep disturbance in PD (31, 32).

In several controlled studies, dopamine agonists (DAs) improved sleep in various aspects, including overall sleep quality, nocturnal disabilities, and sleep maintenance (33–35). However, the use of DAs failed to demonstrate a significant correlation with PDSS-2 in our study. One possible explanation is the administration of a relatively lower dose of dopamine agonists (<1 mg/day on average using pramipexole as an example) to our participants compared with the aforementioned studies. Furthermore, DAs seem to have a two-sided effect to sleep architecture in patients with PD. DAs have been shown to improve sleep quality, nocturnal motor functions and reduce the frequency and severity of REM-sleep behavior disorders in PD in several studies conducted in different ethnics including Asian and Caucasian (36–38). DAs are used to treat restless legs syndrome and periodic limb movements during sleep and are found to reduce the incidence of nocturia (39, 40). However, DAs have been implicated in sleep attacks, sleep maintenance problems, sleep disordered breathing, and hallucinations in patients with PD (41–44). Future studies testing DAs in different doses and different types of sleep disorders are warranted to clarify the role of DAs in PD patients with sleep disorder.

Our study demonstrated that participants who were currently working were more likely to be good sleepers. Among the good sleepers, 57% were currently working. By contrast, only 39% of the poor sleepers were currently working. This can be attributed to the physical exertion caused by regular work, and the potential tendency of participants capable of working to exhibit milder PD symptoms. In the literature, PD has been reported to affect the working capacity with longer disease durations and more severe symptoms (45, 46). In a systemic literature review, retirement of patients with PD were found 4–7 years earlier than general population and was associated with high social costs and loss of individual earnings (47). Anxiety, slowness and fatigue was reported associated with unemployment; (47, 48) these factors also may account for poor sleep quality. On the other hand, continued employment can establish routines which can regulate sleep, prevent lethargy and depression. Such influences can provide an enriched personal and social environment that stimulates positive neuroplasticity and promotes neurocognitive reserve in patients with PD. Thus, working is important both for general life satisfaction and help reducing the symptoms of PD, which may also improve sleep quality (13, 14).

## Conclusion

Poor sleepers constituted approximately half of our patients with PD. Participants experienced more favorable sleep if they were currently working. Increased PD duration, severity, depression or anxiety symptoms, and doses of dopaminergic therapy were significantly associated with poor sleep quality. Continued working, attempts to treat comorbid anxiety or depression, and avoidance of overdosage of dopaminergic treatments may improve sleep quality in patients with PD. The sample size of our study is relatively small; a multicenter, large sample size cohort will identify more precise predictors for sleep quality for patients with PD.

## Author Contributions

C-WC and B-LC: manuscript writing. J-YF: data analysis and manuscript writing. Y-RW: study planning, data analysis, and manuscript writing.

### Conflict of Interest Statement

The authors declare that the research was conducted in the absence of any commercial or financial relationships that could be construed as a potential conflict of interest.
